# A Multi-disciplinary and Comparative Approach to Evaluating Pre-trial Detention Decisions: Towards Evidence-Based Reform

**DOI:** 10.1007/s10610-022-09510-0

**Published:** 2022-05-13

**Authors:** Mandeep K. Dhami, Yannick N. van den Brink

**Affiliations:** 1 Department of Psychology, Middlesex University, The Burroughs, Hendon, London, NW4 4BT UK; 2 Department of Criminal Law and Criminology, Vrije Universiteit Amsterdam, De Boelelaan 1105, 1081HV Amsterdam, The Netherlands

**Keywords:** Pre-trial detention, Bail, Remand, Custody, Courts, Judges, Legal decision-making

## Abstract

The decision to remand a defendant into custody pre-trial is one of the most controversial criminal justice decisions because it deprives individuals of their liberty while they are presumed to be innocent of a crime. Indeed, pre-trial detention decisions can have significant consequences for defendants, which need to be balanced against the potential implications of bail for public safety and the course of criminal proceedings. Despite this, court-based bail and remand decision-making remains relatively underexplored. In this paper, we compare court-based bail/remand decision-making in England and Wales and The Netherlands. We focus on (i) the procedure and structure of decision-making, (ii) the substantive relevant legal frameworks, (iii) the courts in which the decisions are made and the decision-makers in those courts, (iv) the conditions characterizing the decision task, and (v) the court’s reasoning of bail and remand in custody decisions. Using a comparative and multi-disciplinary approach, relying on Law, Criminology, and Psychology, we make predictions about bail and remand in custody rates in the two jurisdictions as well as the decision performance of court-based decision-makers. These predictions are then evaluated using available (official) statistics and past research. We identify the need to collect more nuanced statistical data on bail and remand in custody rates and point to potentially fruitful avenues for future research. A comparative, multi-disciplinary, evidence-based approach can underpin remand reform in England and Wales, The Netherlands, and beyond.

## Introduction

The use of pre-trial detention (i.e., pre-trial remand in custody) is an issue of concern in many jurisdictions throughout Europe (Fair Trials International, 2017; Hammerschick et al., 2017). This is true for England and Wales (EW) and The Netherlands (NL), the two jurisdictions that are central to this article. Remarkably, however, the process of how court-based remand decisions are made is still largely underexplored.

The pre-trial detention decision is arguably one of the most controversial decisions made in the criminal justice system because it deprives defendants of their liberty while they are awaiting trial and when they are to be presumed innocent (Trechsel, 2005). Yet, courts make these decisions on a daily basis—in 2019, there were 29,570 “untried” prison admissions (i.e., pre-trial remands in custody) in EW (Ministry of Justice, 2020d) and 13,965 in NL (WODC et al., 2020).^1^ Remand detainees account for roughly 12% of the overall adult prison population in EW (Ministry of Justice, 2020d) and 45% in NL (WODC et al., 2020).

The remand decision can have significant consequences for defendants. Those who are remanded into custody are more likely to lose their homes, jobs, and contact with their families than those who are bailed (Gibbs, 2018; HM Inspectorate of Prison, 2012; Schönteich, 2008). Pre-trial custody can bias the progress of a case such that defendants remanded into custody are more likely to plead guilty or be convicted at trial and more likely to receive a custodial sentence than their bailed counterparts (Cape & Smith, 2016; Stevens, 2010; Williams, 2003). Remand prisoners are also more likely to commit suicide in prison relative to other prison populations (Ministry of Justice, 2020c; Schönteich, 2008). The remand prison population also poses a resource burden and existential challenge to a penal system whose primary goal is to deal with convicted offenders, not those who may be innocent (HM Inspectorate of Prisons, 2012).

The ramifications of the pre-trial detention decision for individual defendants need to be balanced against the potential implications for victims of crime and wider society. Defendants who are bailed before conviction may abscond, offend, or obstruct justice (e.g., by interfering with witnesses) and so pose a risk to the public and undermine the justice process. For instance, in 2019, 71,000 failure to appear (in court) arrest warrants were issued in EW (Ministry of Justice, 2020b), and in 2014, 36,035 offenders in EW were convicted of an offence committed while on bail (Ministry of Justice, 2015).

Different jurisdictions have sought to solve the problem of what to do with defendants who are charged with a criminal offence and are awaiting trial. In the present paper, we examine how court-based bail/remand decisions are made in EW and NL. We consider the implications that cross-national differences between EW and NL may have for pre-trial bail and detention rates as well as for what we call the “decision performance” of court-based decision-makers. Our definition of decision performance is borrowed primarily from Social Judgment Theory (Hammond et al., 1975). This meta-theory provides a framework to guide research on human judgment in order to improve it (see also Dhami & Belton, 2017).^2^ For present purposes, decision performance refers to the amount and nature of information used as well as how it is weighted and integrated, the extent of intra- and inter-individual variability in decision-making, and the degree of self-insight into one’s decision processes. A comparative and multi-disciplinary approach, grounded in Law, Criminology and Psychology, can advance our understanding of bail/remand decision-making and identify opportunities for future research that can inform evidence-based reform.

## Remand Decision-Making in England and Wales and The Netherlands

In our analysis, we focus on five key areas: (i) procedure and structure of remand decision-making, (ii) the substantive legal frameworks for remand decision-making, (iii) the courts in which initial court-based remand decisions are made and the decision-makers who make them, (iv) the conditions that often characterize the task of remand decision-making, and (v) the court’s reasoning of remand decisions.^3^ In what follows, we compare and contrast EW and NL on each of these and consider the implications for bail and remand in custody rates as well as decision performance. We use available official statistics^4^ and empirical evidence to evaluate these implications.^5^ We also identify the need to collate (statistical) data on specific issues and potential fruitful areas of empirical investigation.

### Procedure and Structure of Remand Decision-Making

In both EW and NL, an individual who is suspected of committing an offence appears in court after he/she has been dealt with by the police and/or prosecution. In both jurisdictions, the laws and policies guiding the decisions of these other bodies are not too dissimilar to those guiding the court’s decisions. The main difference between the two jurisdictions is that in EW, all cases are brought before the remand court, regardless of the police decision to bail (unconditionally or with conditions) or remand in (police) custody. By contrast, in NL, only those cases the police or prosecutor does not decide to bail, i.e., those where a remand in custody is requested by the prosecutor, are brought before the court pre-trial. This “triaging” or “preselection” of cases by the prosecutor in NL implies that judges in NL generally make remand decisions on defendants who are brought to court in custody and that their decision is framed as whether or not to accept the prosecutor’s request to remand a defendant into custody. By contrast, in EW, the courts are presented with the full population of defendants charged with an offence, and they must decide whether or not these defendants should be bailed or remanded into custody.

**Fig. 1:**
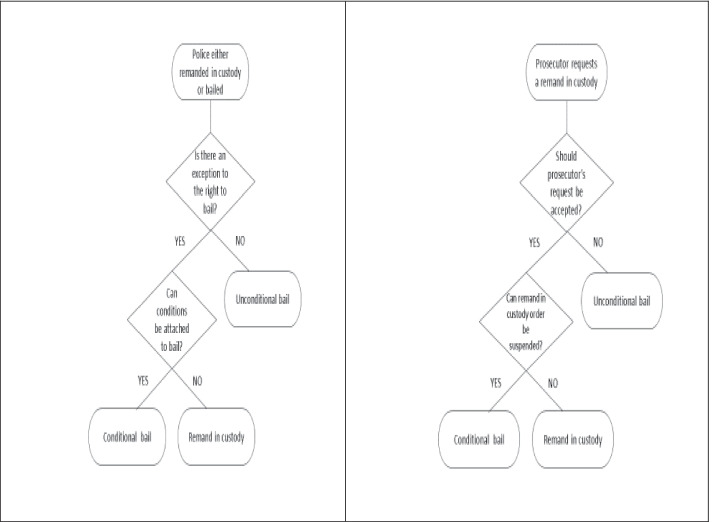
Generalized process of bail/remand decision-making in **a** England and Wales, left panel, and **b** The Netherlands, right panel

Whereas the law on bail in EW provides a general right to bail with exceptions (Sect. 4 of the Bail Act 1976), there is no such right in the relevant Dutch law (see Code of Criminal Procedure 1926 particularly Articles 67 and 67a). Yet, both jurisdictions are party to the European Convention on Human Rights (ECHR), which includes the right to personal liberty (Art. 5) and implies that a remand in custody should only be used in exceptional circumstances (see the European Court of Human Rights’ established case law, e.g., Buzadji v The Republic of Moldova. Consequently, this general rule also formally applies in NL (cf., Zohlandt v The Netherlands), where courts are not only bound by domestic legislation, but also by binding provisions from international treaties such as Article 5 of the ECHR (cf. Arts. 93 and 94 of the Dutch Constitution). Despite this, the fact remains that, due to the different structures provided by domestic remand legislation in EW and NL, the starting point of a court-based remand decision in a case differs across the two jurisdictions. As Fig. 1 illustrates, in EW, the court must decide if there are exceptions to the right to bail, whereas in NL, the judge makes a decision on whether or not to accept the prosecutor’s request to remand a defendant into custody.

The difference between EW and NL in terms of the types of cases brought before the courts and the starting point (or default) for decision-making can have implications for bail and remand in custody rates in the two jurisdictions as well as for the decision performance of court-based bail/remand decision-makers. Decision-makers in EW, faced with the full population of cases which could be bailed or remanded into custody, may be more likely to rely on a wider variety of factors than decision-makers in NL who are faced with a skewed sample of cases. This is because the full population of cases are potentially more variable in terms of their characteristics. However, as will become apparent later, the fact that, unlike in EW, there is a lack of specification in NL of the factors on which risk judgments that inform bail/remand decisions should be based suggests that decision-makers in NL may rely on a broader set of factors than decision-makers in EW.

Psychological research has demonstrated that people tend to choose the default option (i.e., Samuelson & Zeckhauser, 1988) which can save time, effort, and resources (e.g., Carroll et al., 2009; Johnson & Goldstein, 2003). Consequently, remand in custody rates are expected to be greater in NL than EW. However, as we discuss later, the effects on decision performance are confounded or muddied by the unstructured nature of decision-making in NL compared to EW and the paucity of relevant available information to decision-makers during hearings in both jurisdictions. Future research also needs to disentangle the effects of the default option in both jurisdictions from the effects of triaging of cases in NL. Indeed, of the defendants who are brought before the court pre-trial, in NL around 64% are remanded into custody (Algemene Rekenkamer, 2017) compared to approximately 4% in EW (Ministry of Justice, 2020a). Yet, by looking at the overall rate of custodial remands among the total number of cases dealt with (by the prosecutor or court in NL), the difference between the two jurisdictions appears to be negligible—according to official statistics the 2019 remand in custody rate was 5% in NL (WODC et al., 2020) compared to 4% in EW (Ministry of Justice, 2020a).

### The Substantive Legal Frameworks for Remand Decision-Making

There are similarities and differences across the two jurisdictions in terms of the substantive “justifications” that courts can give for a remand in custody based on the law. As mentioned, there are several exceptions to the general right to bail in EW, and exceptions exist for both defendants accused of imprisonable and non-imprisonable offences (Part 1 and 2 of Schedule 1 of the Bail Act 1976).^6^ A defendant may be remanded into custody in EW if there is a risk that the defendant would fail to surrender (abscond), commit an offence on bail, or interfere with witnesses or otherwise obstruct the course of justice. Bail can also be refused for the defendant’s own protection, if the defendant is already serving a custodial sentence, if he/she has been arrested for absconding or breaking bail conditions, if the defendant is accused of a serious offence that it appears was committed while on bail, or if the court deems that there has not been enough time to obtain sufficient information to inform a decision.

In NL, the court’s decision to accept the prosecutor’s request to remand in custody is made on the basis of four legal requirements that all need to be met in a single case (see Arts. 67a and 67a CCP). A first requirement is that a defendant is charged with an offence which carries a maximum custodial sentence of 4 or more years, or charged with a specified offence, although an exception applies for defendants without a permanent residence who are charged with any imprisonable offence. A second requirement is that there are “serious objections” (which refers to the strength of the evidence) against the defendant. A third requirement is that there needs to be one or more lawful grounds (i.e., reasons) that justify remanding a defendant into custody, in short—a risk of absconding, reoffending, and obstructing the process of truth-finding or public disorder. As a fourth requirement, a remand in custody “cannot” be made if there is a “serious account” of the “possibility” that the defendant would not be sentenced to custody (if convicted) or that the period on remand would be lengthier than the duration of an expected custodial sentence.

In both jurisdictions therefore, the main reasons for denying bail (i.e., for a remand in custody) are predominantly risk-based and include, inter alia, the risk of absconding, reoffending, and obstructing justice. In EW, the bail law further states that these risk judgments should be based on a number of specific factors (i.e., the offence and potential sentence, the defendant’s character, prior convictions and community ties, previous bail record, the strength of the evidence), although it also includes a “catch-all” category by stating that in addition to these factors, the court can have regard to “any others which appear to be relevant” (Part 1 of Schedule 1 of the Bail Act 1976). Additionally, in EW the threshold for converting risk judgments into decisions is “substantial grounds.” By contrast, in NL, the law provides very little guidance on making the risk judgments. There, a risk must constitute a “weighty reason related to public safety” in order to justify a remand in custody.

It is clear that the bail/remand law in both jurisdictions is open to subjective interpretation and affords courts considerable discretion in individual cases. For instance, what are the “serious objections” against the defendant in NL and how strong must the evidence against the defendant be in EW? How is “possibility” of a custodial sentence defined in NL and precisely what are the “potential” sentences of concern in EW? What is a “weighty reason” in NL and how “substantial” must the grounds be in EW? Although this can make it difficult to confidently draw a priori predictions about the relative bail and remand in custody rates across the two jurisdictions, some differences in the law suggest that certain types of defendants may be more or less likely to be remanded into custody in one jurisdiction than the other.

First, we would expect a greater proportion of defendants remanded into custody in EW than NL to be facing charges for non-imprisonable offences. Indeed, although in NL specific statistics on the types of charges faced by remand detainees are not publicly available, the law prohibits the use of remand for non-imprisonable offences. There is no such provision in EW. There, in 2019, around 41% of those who were remanded into custody were not charged with violent, drugs, or theft offences (Ministry of Justice, 2020d), although the (non-)imprisonable nature of such offences is not recorded in the available statistics.

Second, we would expect that a greater proportion of defendants to be remanded into custody for their “own protection” in EW than in NL. This is because in EW this ground for denying bail can be applied by magistrates in individual cases, independently of other grounds, while in NL the defendant’s “own protection” is, as such, not recognized by law as a legitimate ground for pre-trial detention. Although we were unable to obtain any statistics to assess this prediction, Cape and Smith (2016) found that decision-makers in EW reported this as a ground they may use for denying bail, and the problem is sufficiently great that efforts are underway to repeal this power (see Howard League for Penal Reform, 2020).

Third, we may expect a large proportion of remand detainees with no permanent address in NL, since under Dutch law, the threshold for using remand is lower when a defendant has no permanent address in the country. Even though we could not find statistics to evaluate this prediction directly, a recent study by Light and Wermink (2020) suggests that foreign nationals (who are expected to comprise a significant proportion of defendants with no permanent address in NL) are less likely to be released pre-trial compared to Dutch nationals. In EW, Cape and Smith (2016) suggest that decision-makers report “no fixed abode” as being a useful consideration in their decision-making (see also Dhami, 2005). A report by HM Inspectorate of Prisons (2012) found that, in EW, 27% of remand prisoners reported having “housing problems” upon entry to prison (see also Howard League for Penal Reform, 2013), and others have similarly documented the likelihood of those with no fixed abode being remanded into custody (e.g., Cooper, 2017).

Fourth, defendants may be more likely to be remanded into custody in NL for risk of reoffending because, unlike in EW, no explicit criteria are required to justify the judged risk. Evidence suggests that in NL, the risk of reoffending accounts for an estimated 80–95% of remand orders (College voor de Rechten van de Mens, 2017; Crijns et al., 2016), and research indicates that judges tend to interpret this ground for remand in custody rather widely (College voor de Rechten van de Mens, 2017; Crijns et al., 2016; Stevens, 2012; Van den Brink, 2018). In EW, research estimates that risk of reoffending may underlie a remand in custody decision in 50–61% of cases (Cape & Smith, 2016; see also Dhami, 2005).

Finally, we may expect that a remand in custody might be a stronger predictor for a custodial sentence in NL than in EW. Under Dutch law, remands in custody cannot be made if it is anticipated that the defendant, if convicted, would not be sentenced to custody, whereas in EW, the likely sentence is one of several criteria that are used to judge the risks of absconding, offending, or obstructing justice, which subsequently inform bail/remand decision-making. In NL, research indicates that roughly 80–85% of defendants who spent time on remand and who are subsequently convicted receive a custodial sentence (College voor de Rechten van de Mens, 2017; Stevens, 2010). In EW, in 2019, 13% of those who were remanded into custody in the magistrates’ court were sentenced to immediate custody after conviction (and 65% were sent to trial or sentencing at the Crown Court; Ministry of Justice, 2020a).^7^ In the Crown Court, 76% of those who were remanded into custody were sentenced to immediate custody (Ministry of Justice, 2020a).

Beyond the effects on bail and custody rates, the unstructured nature of bail/remand decision-making and the discretion this affords may also have several adverse effects on the decision performance of court-based decision-makers. First, the lack of specification of the factors on which risk judgments should be based in NL suggests that decision-makers there may rely on a broader set of factors (including some potentially unwanted or extra-legal factors) and that they may show greater inter-individual variability in the factors they use than decision-makers in EW. Research in EW shows that the majority of decision-makers rely on the same (legal) factors when judging the risk of absconding, offending, or obstructing justice, namely previous convictions and bail record, community ties, and offence, respectively (Dhami, 2005). In NL, research suggests that there is great inter-individual variability in judges’ interpretation of the legal grounds for remand—i.e., the risk of absconding, reoffending, collusion, and public disorder (Van den Brink, 2018). It has also been found that the underpinning risk judgments in NL can be informed by a wide variety of legal and extra-legal factors (College voor de Rechten van de Mens, 2017; Van den Brink, 2018; cf. Van den Brink, 2022). Nevertheless, research additionally indicates that, in NL, the decisive element in judgments of the risk of reoffending and risk of public disorder often ultimately boil down to the perceived seriousness of the offence (Stevens, 2012).

The fact that neither jurisdiction specifies how factors ought to be weighted and integrated has led to studies in EW demonstrating the prevalence of simple heuristic decision strategies where both individual decision-makers’ and benches’ bail/remand decisions can be better predicted by decision models that rely on only one factor than models which integrate multiple factors (Dhami, 2003; Dhami & Ayton, 2001). Equivalent research remains to be conducted in NL, although there is ample evidence on the psychology of human judgment and decision-making to suggest that heuristic strategies are commonplace, even among trained expert/professional samples working in consequential domains (see Gigerenzer et al., 2011; Gilovich et al., 2002).

It is perhaps unsurprising that discretion may result in unwanted variation in both risk judgments and decisions. Research in EW has revealed that risk judgments made on the same case can span the whole 0 to 100% scale across individual decision-makers (Dhami, 2010), and worryingly, this variation is greater for cases judged to be more at risk (Dhami, 2005). Similarly, it has been shown that around a third of decision-makers may disagree with the modal decision made on a case (Dhami, 2005; Dhami & Ayton, 2001), and again, worryingly, this variation is greater for cases which are more likely to be remanded into custody (Dhami, 2005). In EW, variability in decisions has been found to be positively predicted by variability in risk judgments of absconding (Dhami, 2005). Equivalent research in NL is lacking.

Similarly, although it is unknown how high or low the “weighty reasons” threshold is in NL, research in EW has found that there is considerable variation (ranging around 90%) in interpretations of “substantial grounds” for denying bail across decision-makers (Dhami, 2010).

Finally, the aforementioned variability observed across decision-makers (often called “disagreement”) can also be observed within a decision-maker (often called “reliability”). In EW, there is evidence that individuals may make different remand decisions on the same case simply presented a few moments later, with statistical measures of such consistency (i.e., Cohen’s Kappa) ranging from 0.60 to 0.69 out of a maximum of 1 (Dhami, 2002; Dhami & Ayton, 2001). The degree of intra-individual reliability in decision-making in NL remains to be established.

### Remand Courts and Decision-Makers

Another set of main differences between the two jurisdictions refer to the type of court in which the initial court-based bail/remand decisions are made and the characteristics of the decision-makers in these courts as well as their formal roles according to the system’s legal tradition. In EW, the majority of initial court-based bail/remand decisions are made in the lower tier magistrates’ courts. The vast majority of decisions in the magistrates’ court are made by lay magistrates.^8^ They are not required to have any legal qualifications, and they work on an unpaid, part-time basis, making decisions as a bench of two or three, advised on legal matters by a court clerk. By contrast, in NL, an examining (or investigative) judge in a district court makes the first court-based remand decision. These legally qualified judges are paid to make decisions alone, on a full-time basis.^9^

It is reasonable to expect that the decision performance of groups of lay judges working on a part-time basis (representative of EW) and professional judges working on an individual, full-time basis (representative of NL) to be different. Research in EW, however, reveals that there is little difference in the decision performance of lay and professional judges (Dhami & Ayton, 2001) and of lay judges working alone or in groups (Dhami, 2003; Dhami & Ayton, 2001). Years of experience on the bench also has little effect on decision performance (Dhami, 2010; Dhami & Ayton, 2001). As mentioned earlier, decision-makers used simple heuristic strategies and showed concerning levels of (intra-individual) unreliability and (inter-individual) disagreement.

The formal roles of court-based bail/remand decision-makers in EW and NL are different due to the legal traditions of the systems in which they operate (Van den Brink, 2021). Whereas the system in EW is based on common law and is adversarial, NL is a civil law jurisdiction with an inquisitorial criminal justice system. It has been suggested that court-based decision-makers in the former type of system focus more on adhering to procedures rather than truth finding (Findley, 2011/12). They are considered to be passive recipients of information and adjudicate the procedure by which this information is presented to the court. Where opposing parties present conflicting viewpoints, the decision-maker in an adversarial system may be influenced by one side more than the other. By contrast, it is claimed that court-based decision-makers in the latter (inquisitorial) type of system aim to search for truth (Findley, 2011/12). They are viewed as neutral factfinders, managing information and its presentation for the proceedings at hand.

As described in more detail below, research in EW does support the idea that court-based decision-makers are overly influenced by prosecutorial and police requests (Dhami, 2003; Dhami & Ayton, 2001; Grech, 2017; Hucklesby, 1996, 1997; Morgan & Henderson, 1998; Williams, 2000). In an inquisitorial system where the information is not framed in such oppositional terms, the decision-maker may be influenced by a broader set of factors. In NL, however, the prosecutor’s request to remand a defendant into custody is the starting point of the court’s bail/remand decision-making process, which—for the reasons outlined earlier and below—might limit, shape, or even bias the factors that courts take into account when making their decisions (cf. Boone et al., 2019; Crijns et al., 2016; Janssen et al., 2013; Van den Brink et al., 2017).

### Potential Effects of Other Remand Decision Task Conditions

Bail/remand decisions in EW and NL may also be (inadvertently) affected by the conditions under which decisions are made. These task characteristics include the paucity of relevant information available to decision-makers and the fact that the available information is often biased in favor of police or prosecutorial perspectives, problems that have been repeatedly highlighted in both jurisdictions (e.g., Boone et al., 2019; Burrows, 1994; Cape & Smith, 2016; Crijns et al., 2016; Dhami, 2003; Grech, 2017; Hucklesby, 1996; Morgan & Henderson, 1998; Smith, 2020; Williams, 2000). Research evidence in both jurisdictions suggests that bail/remand decisions simply reflect the crime control orientation of the police and prosecution, with decision-makers “rubber-stamping” requests to remand in custody or “passing the buck” by following the decisions of earlier decision-makers such as the police (e.g., Boone et al., 2019; Cape & Smith, 2016; Dhami, 2003; Dhami & Ayton, 2001; Grech, 2017; Hucklesby, 1996, 1997; Janssen et al., 2013; Morgan & Henderson, 1998; Stevens, 2012; Williams, 2000).

Other task characteristics, namely heavy caseloads and the resulting sense of time pressure, have also been frequently documented in both jurisdictions (e.g., Cape & Smith, 2016; College voor de Rechten van de Mens, 2017; Crijns et al., 2016; Dhami, 2003; Grech, 2017; Smith, 2020; Van den Brink, 2022). For example, in an observational study of bail/remand hearings in EW, Dhami (2003) reported that the average duration of hearings was less than 10 min, with some lasting less than 1 min. In NL, an observational study by Crijns et al. (2016) reported that the average length of the initial bail/remand hearing before the examining judge was 24 min (these hearings can include a rather extensive questioning of the defendant by the examining judge), whereas subsequent hearings had an average duration of less than 11 min. The sense of time pressure may partly account for the above mentioned heuristic-based approach to bail/remand decision-making and the disagreement and unreliability in decisions. Psychological research has revealed deleterious effects of time pressure on human information processing (Khoo & Mosier, 2008; Lallement, 2010; see also Maule & Edland, 1997).

### Reasoning of Remand Decisions

Finally, both jurisdictions have in place what we call “checks and restraints” on decisions to remand in custody. Perhaps one of the most well-known is the requirement that decision-makers should provide a rationale for their decision. The European Court of Human Rights emphasizes that “only a reasoned decision can effectively demonstrate to the parties that they have been heard and make appeals and public scrutiny of the administration of justice possible” (Zohlandt v The Netherlands, para. 58). In both EW and NL, providing reasons is not a surefire safeguard against biased, poorly reasoned, or inconsistent decisions.

In EW, the court is required to record and provide the defendant with reasons for denying bail or attaching conditions to bail, and a decision to remand in custody must be accompanied by the exception to the right to bail and the reason for its application (Sect. 5, Subsections 1 to 4 of the Bail Act 1976). In NL, the court is similarly required to provide oral and/or written justifications for accepting or rejecting the prosecutor’s request for a remand in custody and for accepting or denying a defendant’s application for conditional bail, i.e., the suspension of an accepted prosecutorial request to remand in custody (Arts. 24(1) and 78(2) CCP). Researchers examining both jurisdictions have commented that in practice, the court’s reasoning is not always well articulated (e.g., Cape & Smith, 2016; College voor de Rechten van de Mens, 2017; Crijns et al., 2016; Grech, 2017; Raine & Wilson, 1994; 1995; Van den Brink, 2018; Williams, 2000). The European Court of Human Rights recently condemned NL’s failure to ensure that remand decisions are sufficiently reasoned in three separate cases, suggesting that this is a systemic issue (Hasselbaink v The Netherlands; Maassen v The Netherlands; Zohlandt v The Netherlands; see Crijns & Van den Brink, 2021). In NL, the court’s formal reasoning may be quite literally a “tick-the-box” exercise through the use of standardized forms (College voor de Rechten van de Mens, 2017). The situation in EW is not too dissimilar (Cape & Smith, 2016), despite the Law Commission (1999, p. 90) recommending that “The reasons which a court gives for denying bail should explicitly deal with the facts of the individual case, not simply state a recognised relevant consideration or a circumstance pertaining to the accused, without going further and explaining fully why it is necessary to detain the defendant.” The perfunctory approach also does not enable articulation of differences in the reasoning of individual magistrates in EW deciding as a group. Moreover, research in both jurisdictions indicates that it is fairly easy to formulate a reason that falls within the law but which did not actually inform the decision and to hide the real reason for why some defendants are denied bail (Cape & Smith, 2016; Janssen et al., 2013; Stevens, 2012; Van den Brink, 2018).

The provision of reasons may actually be a moot subject. According to psychological research, it may be unrealistic to expect decision-makers to accurately articulate their reasoning (e.g., Brookhouse et al., 1986; Nisbett & Wilson, 1977; Summers et al., 1970). It is difficult for people to fully introspect on the workings of their cognitive system, and even if this were possible, they may not have the necessary language to provide a detailed description of their cognitive operations, and even they did, they may not want to divulge this information for social desirability and self-protective purposes. Dhami and Ayton (2001) found a predictable discordance between the factors that individual decision-makers in EW reported influenced their bail/remand decisions and the factors that predicted their decision behavior. “Legal” factors were ranked higher than “extra-legal” factors in self-reports, whereas the opposite was true in the behavior predicting models. These findings not only have implications for our understanding of so-called checks and restraints on decision-makers, but also warn against relying on self-report data when researching bail/remand decision-making.

## Conclusions

In the present paper, using a comparative and multi-disciplinary perspective, we have considered how bail and remand in custody rates in EW and NL as well as the decision performance of court-based bail/remand decision-makers operating in these two jurisdictions may be affected by a host of factors. These include those related to (i) the procedure and structure of bail/remand decision-making, (ii) the substantive relevant legal frameworks, (iii) the courts in which pre-trial detention decisions are made and the decision-makers working in these courts, (iv) the conditions characterizing the decision task, and (v) the court’s reasoning of pre-trial detention decisions. Where possible, we have used the available official statistics on bail and remand in custody rates and past research on bail/remand decision-making to assess our hypotheses and found that the extant evidence largely supported our predictions. Even though we have presented several indicators which seem to suggest that reliance on pre-trial detention is likely to be higher in NL than EW, the available data and research are currently insufficient to provide conclusive evidence in support of this hypothesis. There are gaps in the official statistics on bail/remand outcomes and the empirical research on bail/remand decision-making in both jurisdictions. The statistical data need to be more nuanced. In particular, the statistics ought to distinguish between bail/custody rates for different types of defendants (cases) as well as capture the specific reasons given for pre-trial detention. For instance, what are the bail/custody rates for defendants charged with imprisonable and non-imprisonable offences in EW, as well as defendants who do or not have a fixed address? What proportion of defendants are remanded into custody for their “own protection”?

Following the psychological research on bail/remand conducted by Dhami, research in NL also needs to examine the (cognitive) decision-making process of the examining judge, measure judges’ interpretations of the “weighty reasons” threshold, and establish the degree of intra- and inter-individual variability in decision-making. Other avenues for future research include examining the effect of experiencing either the population of cases that could be bailed or jailed pre-trial (as in EW) or a skewed sample of cases (as in NL). Research could also directly examine the effect of default options and status quo bias on bail/remand decision-making—although there is ample evidence for these phenomena in other domains, it is prudent to examine their generalizability to the present domain.

Given the inherently controversial nature of pre-trial detention—i.e., the deprivation of liberty of defendants who are to be presumed innocent—and the far-reaching consequences these decisions may have for defendants, victims, and the wider public, as well as the resource burden and existential challenge the remand prison population poses on the prison system, it is striking that an evidence-based approach to pre-trial detention is largely absent in EW and NL. Collation of more detailed statistics on bail/remand in custody rates and research on the factors affecting decision performance can together be used to shape an evidence-based approach to informing and targeting major reforms to bail/remand decision-making in both jurisdictions, who in the past have arguably merely tinkered with the system in a patchwork manner. We hope that this article sparks further multi-disciplinary and comparative research on pre-trial detention and convinces policymakers and practitioners of the need for evidence-based remand reform in EW, NL, and beyond.

## Footnotes

1 We report pre-COVID-19 statistical data on bail and remand in custody rates for the two jurisdictions in order to avoid presenting potentially unrepresentative data because the effects of the pandemic on patterns and trends have yet to be known.

2 Typically, social judgment theorists study experts/professionals making decisions over several trials under conditions of uncertainty and facing multiple fallible items of information. The data are then analyzed for each individual using modeling techniques (e.g., regression statistics), and the findings are used to inform policy and practice (for a review, see Dhami and Mumpower, 2018).

3 Other aspects relevant to bail/remand decision-making such as custody time limits and possibilities of appeal against decisions fall outside the scope of this article, as do decisions about bail conditions.

4 The statistics for EW primarily refer to those published by the Ministry of Justice and which are based on data that has undergone quality assurance (for more detail about sources and quality see https://assets.publishing.service.gov.uk/government/uploads/system/uploads/attachment_data/file/962282/criminal-justice-statistics-guide.pdf and https://assets.publishing.service.gov.uk/government/uploads/system/uploads/attachment_data/file/562956/Guide_to_Offender_Management_Statistics.pdf). The official statistics for NL are, if not otherwise indicated, based on data provided by the Netherlands Court of Audit (*Algemene Rekenkamer*), the Research and Documentation Centre of the Ministry of Justice (WODC), the National Statistical Office (CBS), and/or the Judicial Office (*De Rechtspraak*). For more detail about sources and quality see: https://www.rekenkamer.nl/publicaties/publicaties/2017/11/14/bijlage-voorarrest and https://www.rijksoverheid.nl/documenten/rapporten/2020/10/27/tk-bijlage-criminaliteit-en-rechtshandhaving-2019.

5 Empirical evidence regarding EW comes from research involving quantitative (e.g., Dhami, 2003, 2005, 2010; Dhami & Ayton, 2001) and combined quantitative and qualitative (e.g., Cape & Smith, 2016; Hucklesby, 1996; Williams, 2000) analyses of bail/remand decisions. The data for this research was collected using surveys (e.g., Cape & Smith, 2016; Dhami & Ayton, 2001; Hucklesby, 1996) and interviews (e.g., Cape & Smith, 2016; Hucklesby, 1996; Morgan & Henderson, 1998; Williams, 2000) with decision-makers and other relevant groups such as prosecutors, observations of court bail/remand hearings (e.g., Cape & Smith, 2016; Dhami, 2003; Hucklesby, 1996; Morgan & Henderson, 1998; Williams, 2000), examination of case files (e.g., Cape & Smith, 2016; Hucklesby, 1996), and experimentation with decision-makers (e.g., Dhami, 2005, 2010; Dhami & Ayton, 2001). Research on NL similarly comes from involving quantitative (e.g., Light & Wermink, 2020; Van den Brink et al., 2017), qualitative (e.g., Boone, Jacobs, & Lindeman, 2019; Stevens, 2012; Van den Brink, 2018), and combined (e.g., Crijns, Leeuw, & Wermink, 2016; Stevens, 2010) analyses of bail/remand decisions based on surveys (e.g., Crijns, Leeuw, & Wermink, 2016) and interviews (e.g., Boone, Jacobs, & Lindeman, 2019; Crijns, Leeuw, & Wermink, 2016; Stevens, 2012; Van den Brink, 2018) with decision-makers and other relevant groups, observations of bail/remand court hearings (e.g., Boone, Jacobs, & Lindeman, 2019; Crijns, Leeuw, & Wermink, 2016; Van den Brink, 2018), and examination of case files (e.g., Boone, Jacobs, & Lindeman, 2019; College voor de Rechten van de Mens, 2017; Crijns, Leeuw, & Wermink, 2016; Van den Brink et al., 2017).

6 The courts in both EW and NL can apply any variety and number of conditions to bail (for more on this, in EW see Dhami (2004), and for NL, see Crijns, Leeuw, and Wermink (2016)).

7 In EW, the magistrates’ court deals with the vast majority of criminal cases, while the Crown Court deals with a minority of cases (i.e., most serious ones). Both courts can pass custodial sentences, although the maximum length is restricted in the magistrates’ court to 6 months’ for a single offence (or concurrently, if sentencing for more than one offence), whereas there are no such limits in the Crown Court, beyond the maximum penalty associated with an offence.

8 A much smaller minority of decisions in the magistrates’ courts are made by district judges (previously known as stipendiary magistrates) who are legally qualified, work on a full-time, paid basis, and make decisions alone.

9 In NL, subsequent remand decisions on defendants before conviction are made by two other sets of judges, both of which are legally qualified, work on a full- or part-time basis, and make decisions as a group of three or alone. For present purposes, the predicted effect of judge characteristics on decision-making will be based on the characteristics of the examining judge.

## Declarations

### About the authors

Mandeep K. Dhami, PhD, is Professor of Decision Psychology at Middlesex University, London. Mandeep is an expert on human judgment and decision-making, risk perception, and uncertainty communication. She conducts research on these issues in the criminal justice and intelligence analysis domains. Mandeep is currently Editor of *Judgment and Decision Making*. Yannick N. van den Brink, PhD, is Associate Professor of Criminal Law and Criminal Procedure at Vrije Universiteit Amsterdam, Department of Criminal Law and Criminology, the Netherlands, and a Rubicon Research Fellow at University of Cambridge, Institute of Criminology, United Kingdom. Van den Brink also serves as a deputy judge in the Youth Court of the District Court of The Hague. He coauthored this article in his capacity as an academic researcher.
